# Technology Use Among Cancer Patients Pre- and Post- COVID-19 Pandemic: The Role of Dementia

**DOI:** 10.1093/geroni/igab046.2584

**Published:** 2021-12-17

**Authors:** Weixin Li, Katherine Ornstein, Yan Li, Bian Liu

**Affiliations:** 1 Icahn school of Medicine at Mount Sinai, Long island city, New York, United States; 2 Icahn School of Medicine at Mount Sinai, New York, New York, United States; 3 Icahn School of Medicine at Mount Sinai, Icahn School of Medicine at Mount Sinai, New York, United States

## Abstract

COVID-19 has highlighted increasing reliance on information and communication technology (ICT) and challenges in access and use. ICT access also provides resources that benefit users’ mental health. Our study describes changes in the use of ICT before and during the COVID-19 pandemic among cancer patients with and without dementia. We identified 196 (1.6 million weighted population) older adults with a self-reported cancer history who participated in both 2019 and 2020 National Health and Aging Trends Study (NHATS). In 2019, cancer patients with dementia (9.9%) were less likely (adjusted OR 0.29; 95%CI, 0.11-0.78) to use information technology (IT) for health matters (contacting medical providers, handling health insurance matters, obtaining information about health conditions, and ordering prescription refills) compared to those without dementia. In contrast, dementia status was not associated with communication technology (CT) use (email or texts) or IT use for personal tasks (grocery shopping or online banking). IT use for personal tasks was inversely associated with anxiety symptoms (adjusted OR 0.22; 95%CI:0.06-0.83) and CT use was inversely associated with depressive symptoms (adjusted OR 0.25 (95%CI:0.07-0.97). In 2020, regardless of dementia status, all cancer patients increased their virtual (email/phone/video) contact with family, friends (3.4%-7.0%), and medical providers (17.2%-36.2%) while decreasing in-person contact (10.0%-15.7% and 21.8%-24.2%, respectively) during the pandemic. This study suggests that there are potential unmet daily needs for patients with comorbid cancer and dementia that may be met with improved ICT access. Such challenges are of increasing concern as COVID-19 has resulted in increased ICT reliance for older adults.

